# Specificity of scattering of ultrashort laser pulses by molecules with polyatomic structure

**DOI:** 10.1038/s41598-022-09134-8

**Published:** 2022-03-23

**Authors:** D. N. Makarov, K. A. Makarova, A. A. Kharlamova

**Affiliations:** Laboratory of Diagnostics of Carbon Materials and Spin-Optical Phenomena in Wide-Bandgap Semiconductors, Northern (Arctic) Federal University, Arkhangelsk, Russia 163002

**Keywords:** X-rays, Atomic and molecular interactions with photons, Attosecond science

## Abstract

The theory of scattering of ultrashort laser pulses (USP) is the basis of diffraction analysis of matter using modern USP sources. At present, the peculiarities of interaction of USP with complex structures are not well developed. In general, the research focuses on the features of the interaction of USP with simple systems, these are atoms and simple molecules. Here we present a theory of scattering of ultrashort laser pulses on molecules with a multi-atomic structure, taking into account the specifics of the interaction of USP with such a substance. The simplicity of the obtained expressions allows them to be used in diffraction analysis. As an example, the scattering spectra of deoxyribonucleic acid (DNA) and ribonucleic acid (RNA) are presented. It is shown that the theory developed here is more general in the scattering theory and passes into the previously known one if we consider the duration of the USP to be sufficiently long.

## Introduction

X-ray scattering is at the heart of X-ray structural analysis (XRD)^1–4^. Similarly for X-ray ultrashort pulse (XRD) scattering on matter. XRD is one of the most important methods to study the structure and properties of matter, which is based on the use of X-ray diffraction. The structures of most crystals and many molecules have been determined with this method and underlie many modern discoveries in physics, chemistry, biology, medicine and crystallography, such as^2^. At present, special attention is paid to the physics of ultrashort pulses^5–7^ by creating new types of radiation sources, increasing the power and reducing the duration of ultrashort pulses, etc.^5–9^. Using such USP, it is possible to conduct research on the structure of matter with high temporal and spatial resolution. The more so now there is a technical possibility to conduct such studies. One of the most promising sources of USP are free-electron XFELs^10^. At present, the formation of attosecond pulses is reported due to improvements in X-ray free-electron lasers techniques^11,12^. Here also reached the subfemtosecond barrier with high peak power, which allows to study the excitation in the molecular system, the movements of valence electrons with high temporal and spatial resolution, for example^13^. Due to the creation of high-power USP sources, there is a need for new theoretical approaches that take into account the specifics of the interaction of such USP with complex polyatomic structures^9,14^.

It is well known that the theory of X-ray diffraction by various periodic and complex structures is based on the scattering of plane waves of infinite duration in time^15^. The same theory is used to analyze and decipher the structures of various objects using USP. Scattering processes with femto- and especially attosecond time resolution on such structures have not been studied enough and are being actively developed nowadays^9,16–25^. Usually such theories consider simple systems such as atoms, simple molecules, model systems, systems of one-part atoms, etc. A simple enough theory, which takes into account the specificity of X-ray scattering on complex polyatomic structures, currently does not exist. For example^20^, developed the USP scattering theory in the general case, where there are no restrictions on the number of atoms in the scattering system. Although the expressions obtained in this theory are general and do not allow one to directly calculate the scattering spectra for complex multiatomic systems. In the work^26^, the wave function of atomic and molecular electrons in the USP field was found. In the articles^21,22^, the USP scattering theory was developed taking into account the first and second harmonics on the simplest polyatomic systems consisting of atoms of the same kind, while using the works^20,26^. In^27^, the theory of USP scattering was developed, but not without taking into account the specifics of USP scattering on complex polyatomic systems.

In the present paper, such a theory, obtained on the basis of the sudden perturbation approximation, will be presented. The results will have a simple analytical form and can be applied to calculations of scattering spectra for complex polyatomic structures. It is shown that the well-known theory of X-ray diffraction analysis (XRD) may have errors when used for attosecond pulses. As an example, the case of scattering of X-ray USP on nucleotides (adenine, guanine, cytosine, thymine), which are the basis of deoxyribonucleic acid (DNA), will be considered. It is shown that the scattering spectra are sensitive to spatial changes in the position of atoms in nucleotide structures. The results obtained can be easily extended to more complex structures, including deoxyribonucleic (DNA) and ribonucleic (RNA) acids.

Next, we will use the atomic system of units: $\hbar$ = 1; |*e*| = 1; $m_e$ = 1, where $\hbar$ is the Dirac constant, *e* is the electron charge, $m_e$ is the electron mass.

## Specifics of scattering of X-ray ultrashort pulses

Consider a molecule with a complex polyatomic structure. USP falls on this molecule in the $\mathbf{n}_0$ direction. We assume that the duration of such a pulse $\tau$ is many times less than the characteristic atomic time $\tau _a \sim 1$, i.e. $\tau \ll \tau _a$. It is well known that this condition is applicable in the sudden perturbation approximation. In the sudden perturbation approximation, the intrinsic Hamiltonian of the system can be neglected, since the electron in the atom does not have time to evolve under the action of the USP field^26^. The $\tau \ll \tau _a$ condition can be extended for X-ray USPs and consider that the sudden perturbation approximation is applicable at $\omega _0 \tau _a \gg 1$, where $\omega _0$ is the carrier frequency of the incident USP^22,26^. Further, we will use the USP electromagnetic field strength in the general form $\mathbf{E}(\mathbf{r},t)=\mathbf{E}_0 h (t-\mathbf{n}_0\mathbf{r}/c)$, i.e. we will consider it spatially inhomogeneous, where $\mathbf{E}_0$ is the field amplitude, and $h (t-\mathbf{n}_0\mathbf{r}/c)$ is an arbitrary function defining the USP form, *c* is the speed of light (in a.u. $c\approx 137$). In the case of such pulses, in^26^, when solving the Dirac equation, the wave function of an electron in the USP field with a strength $\mathbf{E}(\mathbf{r},t)$ was found, which we will use below. We will consider the fields not so strong as to account for the magnetic field of the USP, i.e., we will assume that $\mathrm{E}_0/c^2 \ll 1$ or in units of intensity $\mathrm I\ll 10^{25}W/sm^2$. In this case, as shown in^26^, the wave function of a complex multi-electron system can be represented as

1 $$\begin{aligned} \Psi (t)=\varphi _{0}(\{\mathbf{r}_a\})e^{-\sum \limits _{a}i\int ^{t}_{-\infty } \mathbf{E}(\mathbf{r}_a,t')\mathbf{r}_a d t' }, \end{aligned}$$

where $\sum _a$ is the summation over all electrons in a complex polyatomic structures, $\varphi _{0}(\{\mathbf{r}_a\})$ is the initial wave function of all electrons in such a system.

To calculate the basic scattering characteristics, we will use the quantum theory of USP scattering, in which there are no restrictions on the number of atoms in the system^20^. In this theory, general expressions for calculations of the main scattering characteristics are derived. As a result, using Eq. (1) and the theory in^20^ we obtain an expression to calculate the scattering energy $\varepsilon$ per unit solid angle $\Omega _\mathbf{k}$ ($\mathbf{k}=\frac{\omega }{c} \mathbf{n}$, where $\mathbf{n}$ is the direction of the scattered pulse) in the unit frequency interval $\omega$ (hereafter the spectrum)

2 $$\begin{aligned} \frac{d^2\varepsilon }{d\Omega _\mathbf{k}d\omega }= \frac{\left[ \mathbf{E}_0\mathbf{n}\right] ^2}{(2\pi )^2} \frac{|\tilde{h}(\omega )|^2}{c^{3}}\left \langle \varphi _{0} \mid \sum \limits _{a,a^{'}} e^{-i\mathbf{p(\mathbf{r}_a-\mathbf{r}_{a^{'}})}} \mid \varphi _{0} \right\rangle , \end{aligned}$$

where $\tilde{h}(\omega )=\int \nolimits _{-\infty }^{+\infty } h(\eta ) e^{i \omega \eta } d\eta$, and $\mathbf{p}=\frac{\omega }{c} (\mathbf{n}-\mathbf{n}_0)$ has the meaning of recoil momentum when a USP is scattered on a bound electron. Next, we use the well-known model of independent atoms, see for example^22,27^. In this case, the problem can be solved by passing to the electron density of individual isolated atoms that make up a complex polyatomic structure. Dividing the Eq. (2) by two, where the first term corresponds to the summation at $a=a^{'}$, and the second term at $a\ne a^{'}$, we obtain

3 $$\begin{aligned} \frac{d^2\varepsilon }{d\Omega _\mathbf{k}d\omega }= \frac{\left[ \mathbf{E}_0\mathbf{n}\right] ^2}{(2\pi )^2} \frac{|\tilde{h}(\omega )|^2}{c^{3}} \Biggl [ \sum ^s\limits _{i=1} N_{e,i} N_{A,i}(1-|F_{i}|^2)+ \sum ^s\limits _{i,j=1}\delta _{i,j} N_{e,i} N_{e,j}F_{i}F^*_{j}\Biggr ] \end{aligned}$$

where $N_{e,i}$ is the number of electrons in the atom *i* variety; $N_{A,i}$ is the number of atoms *i* variety; $F_{i}=\frac{1}{N_{e,i}}\int \rho _{e,i}(\mathbf{r})e^{-i\mathbf{p}\mathbf{r}}d^3\mathbf{r}$ is the form factor of the *i* atom of the variety with electron density $\rho _{e,i}(\mathbf{r})$. The factor $\delta _{i,j}=\sum _{Ai,A^{'}j}e^{-i\mathbf{p(\mathbf{R}_{Ai}-\mathbf{R}_{A^{'}j})}}$ depends only on the coordinates of atoms *i* of the variety (with number *Ai*) whose position is determined by the radius vector $\mathbf{R}_{Ai}$. The Eq. (3) is analytic, which contributes to a fairly simple calculation of the spectra. The main difficulty in the calculation is determined by the factor $\delta _{i,j}$, since for complex systems it is difficult to find an analytical expression for it. This factor determines the interference and only in this factor the coordinates of atoms in a complex polyatomic structures are concentrated. For fairly simple systems consisting of a single variety of atoms such a factor has been found for many carbon systems^21,22^: graphene, nanotube, atomic rings, “forest” of nanotubes, etc. Of greatest interest for XRD is the $\tau \omega _0\gg 1$ case ($\tau$ is the pulse duration, $\omega _0$ is the USP carrier frequency). If we assume that $\tau \rightarrow \infty$, i.e. the radiation source is continuous, we get the well-known XRD theory. It is this theory that is used in XRD even in the case of ultrashort pulses, without taking into account the specifics of USP scattering. Let us show that the scattering theory elaborated here may differ from the well-known XRD theory when attosecond pulses are used in the case of $\tau \omega _0\gg 1$. To do this, we integrate the expression (3) with respect to frequency, taking into account that the main part in the integration is concentrated near $\omega _0$. Indeed, choosing the $\tau \rightarrow \infty$ case, it is well known that the pulse form is $h=e^{-i(\omega _0 t-\mathbf{k}_0 \mathbf{r})}$ (plane wave). It is easy to see that in this case $|\tilde{h}(\omega )|^2=2\pi T \delta (\omega -\omega _0)$, where $\delta (\omega -\omega _0)$ is the Dirac delta function, *T* is the time of action of the radiation on the system (in general, $T=C\tau$, where the constant *C* depends on the form of the USP). If $\tau \ne \infty$, then the pulse form $h=e^{-i(\omega _0 t-\mathbf{k}_0 \mathbf{r)}} f(( t-\mathbf{n}_0 { \mathbf{r}})/\tau )$, where the *f* function defines the USP profile. It is easy to see that in this case $\tilde{h}(\omega )= \tau \tilde{f}((\omega -\omega _0)\tau )$ ($\tilde{f}((\omega -\omega _0)\tau )=\int ^{\infty }_{-\infty }e^{-i(\omega -\omega _0)\tau \eta }f(\eta ) d\eta$ is the Fourier transform of the function $f(\eta ))$. In this case, we see that after integration over frequency, Eq. (3) will have the form

4 $$\begin{aligned}&\frac{d\varepsilon }{d\Omega _\mathbf{k}}= \frac{\left[ \mathbf{E}_0\mathbf{n}\right] ^2}{(2\pi )^2 c^3} \tau \int ^{\infty }_{-\infty }|\tilde{f}(x)|^2 dx \Biggl [ \sum ^s\limits _{i=1} N_{e,i} N_{A,i}(1-|F_{i}(\mathbf{p}_0)|^2)+ \sum ^s\limits _{i,j=1}\delta _{i,j}(\mathbf{p}_0)\beta _{i,j}(\mathbf{p}_{\tau }) N_{e,i} N_{e,j}F_{i}(\mathbf{p}_0)F^*_{j}(\mathbf{p}_0)\Biggr ], \nonumber \\&\beta _{i,j}(\mathbf{p}_{\tau }) =\frac{\int ^{\infty }_{-\infty }|\tilde{f}(x)|^2 e^{-i x \mathbf{p}_{\tau } (\mathbf{R}_{Ai}-\mathbf{R}_{A^{'}j})} dx}{\int ^{\infty }_{-\infty }|\tilde{f}(x)|^2 dx}. \end{aligned}$$

where $$F_{i}(\mathbf{p}_0), \delta _{i,j}(\mathbf{p}_0)$$ are the expressions defined above, but with the difference that $$\mathbf{p} \rightarrow \mathbf{p}_0=\frac{\omega _0}{c} 
(\mathbf{n}-\mathbf{n}_0)$$ and $$\mathbf{p}_{\tau }=\frac{1}{c \tau } (\mathbf{n}-\mathbf{n}_0)$$. In Eq. (4) it was taken into account that $$c \tau \gtrsim 1$$. From Eq. (4) one can obtain the well-known expression in XRD for the intensity of the scattered radiation if one considers $$\tau \rightarrow \infty$$. Indeed, for $$\tau \rightarrow \infty$$ the parameter $$\beta _{i,j}(\mathbf{p}_{\tau })\rightarrow 1$$. Also, if we consider a sufficiently large number of atoms in the system, so that we can neglect the first term in Eq. (4), i.e. we will assume that coherent radiation is dominant. In this case, we get

5 $$\begin{aligned} \frac{d^2\varepsilon }{d\Omega _\mathbf{k} d T}= \frac{\left[ \mathbf{E}_0\mathbf{n}\right] ^2}{2\pi c^3} \Biggl |\rho (\mathbf{R})e^{-i \mathbf{p}_{0}{} \mathbf{R}} d^3\mathbf{R} \Biggr |^2, \end{aligned}$$

where $\rho (\mathbf{R})=\sum _{Ai}\int \delta (\mathbf{R}-\mathbf{R}_{Ai}-\mathbf{r})\rho _{e,i}(\mathbf{r})d^3\mathbf{r}$ is the distribution of electron density in a polyatomic system in space, $T=\frac{\tau }{2\pi }\int ^{\infty }_{-\infty }|\tilde{f}(x)|^2 dx$. Eq. (5) is well known in XRD. Thus, in the case of multi-cycle pulses, i.e. when $\tau \omega _0\gg 1$ and the polyatomic system Eq. (4) different from Eq. (5) when $\beta _{i,j}(\mathbf{p}_{\tau }) \ne 1$. This is only possible if $\mathbf{p}_{\tau } (\mathbf{R}_{Ai}-\mathbf{R}_{A^{'}j})$ is not small, those. when we consider pulses with duration $\tau \lesssim 1$ (in a.u.), which are attosecond pulses. It should also be added that differences appear not only for attosecond pulses, but also for sufficiently large polyatomic systems, where $(\mathbf{R}_{Ai}-\mathbf{R}_{A^{'}j}) \gg 1$. Such systems can be various nanosystems, biomolecules, etc.

Next, let’s analyze Eq. (3). Equation (3) takes into account both coherent USP scattering (second term) and incoherent scattering (first term). In the general case, the predominance of the coherent over the incoherent factor is determined by many factors. If the USP is multi-cycle, i.e. $\tau \omega _0\gg 1$ ($\tau$ is the pulse duration, $\omega _0$ is the USP carrier frequency), then coherent scattering prevails over incoherent scattering at $\lambda _0 \gg 1$. In the case of multi-cycle USP, the predominance of the incoherent term over the coherent one, the problem is determined not only by the condition $\lambda _0 \ll 1$ but also by the number of atoms in the system in question. In the case of low-cycle and sub-cycle pulses, it is necessary to consider a particular polyatomic structures and the form of the USP to determine the predominance of coherent over coherent; this cannot be done in a general form.

**Figure 1:**
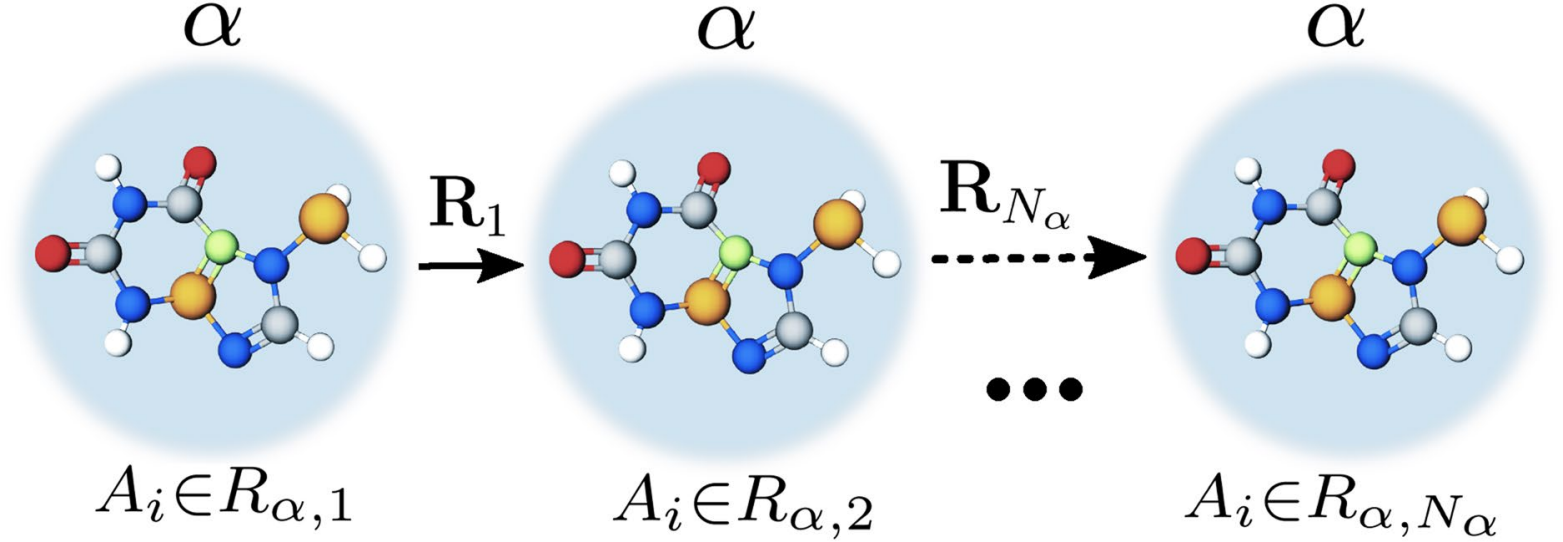
Schematic representation of the main parameters included in the factor $\delta _{i,j}$ calculated by the Eq. (6). Multicolored circles are atoms; one color specifies a certain kind of atoms. If the arrangement of atoms in the system is repeated—first, second, etc. up to $N_{\alpha }$ large circles, this sets the symmetry $\alpha$, and $\mathbf{R}_{1},\mathbf{R}_{2},. \ldots \mathbf{R}_{n_{\alpha }},\ldots , \mathbf{R}_{N_{\alpha }}$ are radius vectors setting the position of $1,2,\ldots , n_{\alpha },\ldots , N_{\alpha }$ large circles. For example, the symmetry with $\alpha =1$ is represented in this figure, and with $\alpha =2$ the location, color, number of circles inside the big circle, number of big circles, and $\mathbf{R}_{n_{2}}$ would be different.

If the polyatomic structures has a certain symmetry or periodicity, it is reflected only in the $\delta _{i,j}$ factor. For polyatomic systems it is difficult to analyze and calculate the scattering spectra during the numerical calculation of Eq. (3). In order to make the calculation and analysis simple, it is necessary for polyatomic structures having a certain symmetry and periodicity, to represent the factor $\delta _{i,j}$ in the analytical form. In general, for such systems the factor $\delta _{i,j}$ can be represented as

6 $$\begin{aligned} \delta _{i,j}=\sum ^s_{\alpha =1}\sum ^{N_{\alpha }}_{n_{\alpha }=0}e^{i\mathbf{p}{} \mathbf{R}_{n_{\alpha }}} \sum _{A_i \in R_{\alpha ,1}}e^{i\mathbf{p}\mathbf{R}_{A_i}} \sum ^s_{\beta =1}\sum ^{N_{\beta }}_{n_{\beta }=0}e^{-i\mathbf{p}{} \mathbf{R}_{n_{\beta }}} \sum _{A_j \in R_{\beta ,1}}e^{-i\mathbf{p}\mathbf{R}_{A_j}}, \end{aligned}$$

where $\alpha$ or $\beta$ is some symmetry in the system, *s* is the number of symmetries in the system, $\mathbf{R}_{n_{\alpha }}$ or $\mathbf{R}_{n_{\beta }}$ is the radius vector that sets the symmetry position $\alpha$ or $\beta$ respectively, $N_{\alpha }$ is the number of translations with a given symmetry, $\mathbf{R}_{A_i}$ is the radius vector specifying the position of the atoms of variety *i* within the region $R_{\alpha ,1}$ (analogously $\mathbf{R}_{A_j}$), see Fig. 1. The importance of Eq. (6) is determined by the fact that it is not necessary to calculate numerically the parameter $\delta _{i,j}$ by summing up all positions of atoms in space. It is enough to determine the symmetry of the object under study and find the sum $\sum ^{N_{\alpha }}_{n_{\alpha }=0} e^{i\mathbf{p}{} \mathbf{R}_{n_{\alpha }}}$ in analytical form. The sum $\sum _{A_i \in R_{\alpha ,1}}e^{i\mathbf{p}{} \mathbf{R}_{A_i}}$ can be found analytically if there is symmetry inside the region $A_j \in R_{\alpha ,1}$ or numerically, where summation is sufficient only in the region $R_{\alpha ,1}$. For example, in the simplest case of a one-atom cubic lattice: $s=\alpha =1$, $i=j=const=1$, $\sum _{A_i \in R_{\alpha ,1}}e^{i\mathbf{p}{} \mathbf{R}_{A_i}} =e^{i\mathbf{p}{} \mathbf{R}_{A_1}}$, since the atom within the symmetry is alone. As a result, for this case the factor $\delta _{1,1}=\bigl |\sum ^{N_{\alpha }}_{n_{\alpha }=0} e^{i\mathbf{p}\mathbf{R}_{n_{\alpha }}}\bigr |^2$, which is well known^21^ and calculated in a simple analytical form.

## Specifics of scattering on DNA nucleotides

As shown above, the USP scattering for polyatomic systems differs from the well-known Eq. (5) for attosecond pulses. Therefore, let’s consider one of the most interesting examples of a polyatomic system - nucleotides: adenine, guanine, thymine and cytosine. Each of these nucleotides forms the backbone of DNA. Each of the nucleotides in the DNA molecule is repeated, which means that there is a symmetry that can be calculated in the factor $\delta _{i,j}$. The most interesting thing is that this symmetry can be modified by modeling the contraction, stretching or twisting of the DNA molecule. The change in symmetry and the symmetry itself should be reflected in the scattering spectra calculated from Eq. (3). Let us calculate the scattering spectra on the following nucleotides separately: adenine, guanine, thymine, and cytosine. In this case, we need to find the factor $\delta _{i,j}$, with $s=1, N_{\alpha }=1$, then $\delta _{i,j} =\sum _{A_i \in R_{1,1}}e^{i\mathbf{p}{} \mathbf{R}_{A_i}} \sum _{A_j \in R_{1,1}}e^{-i\mathbf{p}{} \mathbf{R}_{A_j}}$. These nucleotides are non-periodic and asymmetric systems, so the calculation of the scattering spectrum will be done directly by substituting the coordinates of the atoms in the nucleotide into the factor $\delta _{i,j}$. To calculate scattering spectra we will use the model of independent atoms^28^, in which molecules are represented by independent isolated atoms. The electron density of such atoms $\rho _{e,i}(\mathbf{r})=\frac{N_{e,i}}{4\pi r}\sum ^3_{k=1}A_{k,i} \alpha ^2_{k,i} e^{-\alpha _{k,i} r}$, where $A_{k,i}, \alpha _{k,i}$ are constant coefficients (for all varieties of atoms with number *i*) defined in^28^. The result is a simple expression for $F_i=\sum ^3_{k=1}\frac{A_{k,i} \alpha ^2_{k,i}}{p^2+\alpha ^2_{k,i}}$. Next, we need to determine the form of the incident USP, which we choose as a Gaussian form $h(t)=e^{-i(\omega _0 t-\mathbf{k}_0\mathbf{r})}e^{-\alpha ^2(t-\mathbf{n}_0\mathbf{r}/c)^2}$, where $\alpha = 1/\tau$, $\mathbf{k}_0=\mathbf{n}_0 \omega _0/c$. The Gaussian USP is chosen as one of the best known for describing USP. For example, in^29^ an exact description of the subcyclic pulse beam (SCPB) was found, where in the case considered in this paper ($\omega _0/\alpha \gg 1$) the solution has the form of a Gaussian impulse. In the chosen USP case, we obtain $\tilde{h}(\omega )=\frac{\sqrt{\pi }}{ \alpha }e^{-(\omega -\omega _0)^2/4\alpha ^2}$. Consider the case of multi-cycle momentum, i.e., when $\omega _0/\alpha \gg 1$, which is mainly used in diffraction analysis of matter. Using the expression (4) we obtain

7 $$\begin{aligned} \frac{d\varepsilon }{d\Omega _\mathbf{k}}= \frac{\left[ \mathbf{E}_0\mathbf{n}\right] ^2}{2 c^3 \alpha \sqrt{2\pi }} \Biggl [ \sum ^s\limits _{i=1} N_{e,i} N_{A,i}(1-|F_{i}(\mathbf{p}_0)|^2)+ \sum ^s\limits _{i,j=1}\delta _{i,j}(\mathbf{p}_0)e^{-\frac{1}{2} (\mathbf{p}_{\tau } (\mathbf{R}_{Ai}-\mathbf{R}_{A^{'}j}))^2} N_{e,i} N_{e,j}F_{i}(\mathbf{p}_0)F^*_{j}(\mathbf{p}_0)\Biggr ] \end{aligned}$$

**Figure 2:**
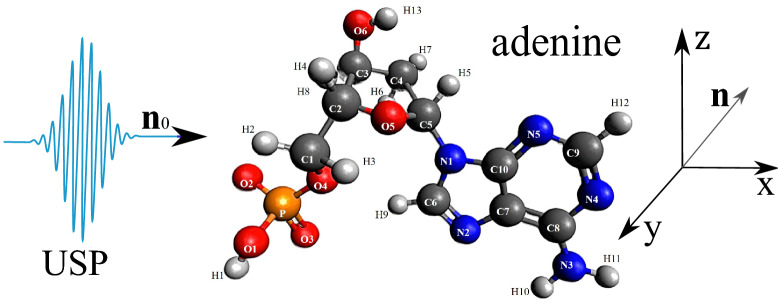
The adenine on which the USP falls is represented, as well as the chosen coordinate system. The calculation was performed in the spatial orientation of the adenine in relation to the USP in the chosen coordinate system shown in this figure.

**Figure 3:**
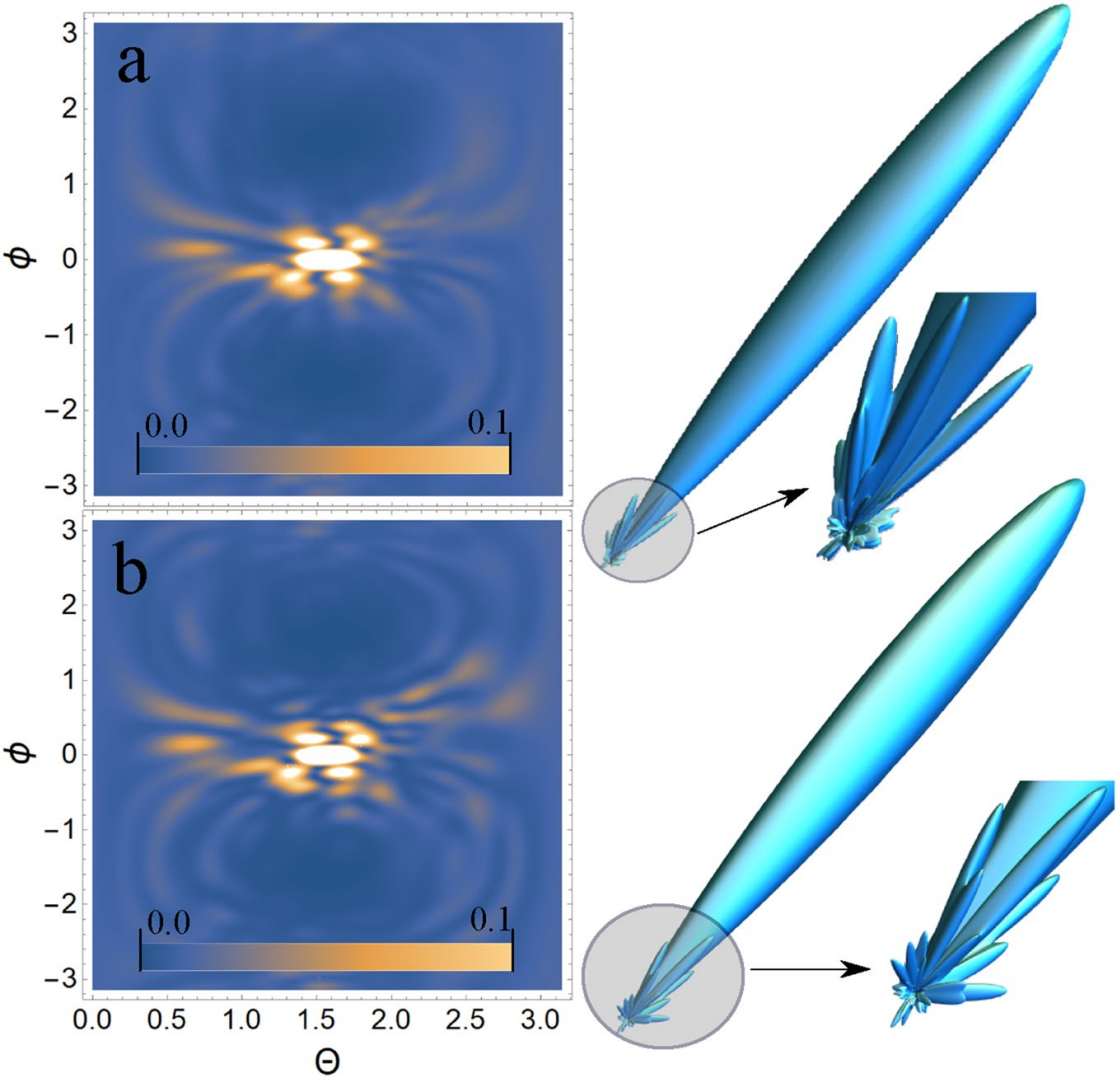
Scattering spectra of USP on adenine are presented. (**a**) Contour plot of the normalized scattering spectrum calculated using Eq. (4) is shown on the left ($\theta$ is the angle between the $\mathbf{n}$ vector and the *z* axis; $\phi$ is the angle between the projection of the vector $\mathbf{n}$ on the *xOy* plane and the *x* axis), and the 3D spatial scattering spectrum with a notch from the region where the scattering is most intense is shown on the right. (**b**) Contour plot of the normalized scattering spectrum calculated using Eq. (5) is shown on the left, and the 3D spatial scattering spectrum with a notch from the region where the scattering is most intense is shown on the right.

**Figure 4:**
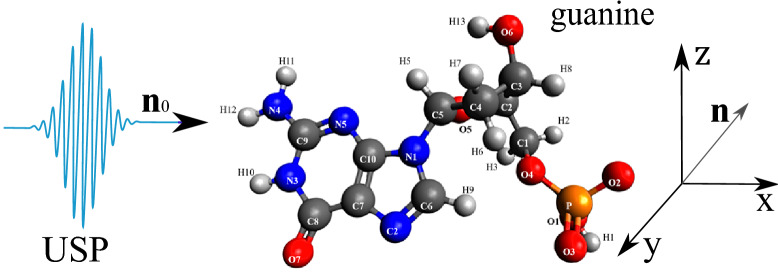
The guanine on which the USP falls is represented, as well as the chosen coordinate system. The calculation was performed in the spatial orientation of the guanine in relation to the USP in the chosen coordinate system shown in this figure.

**Figure 5:**
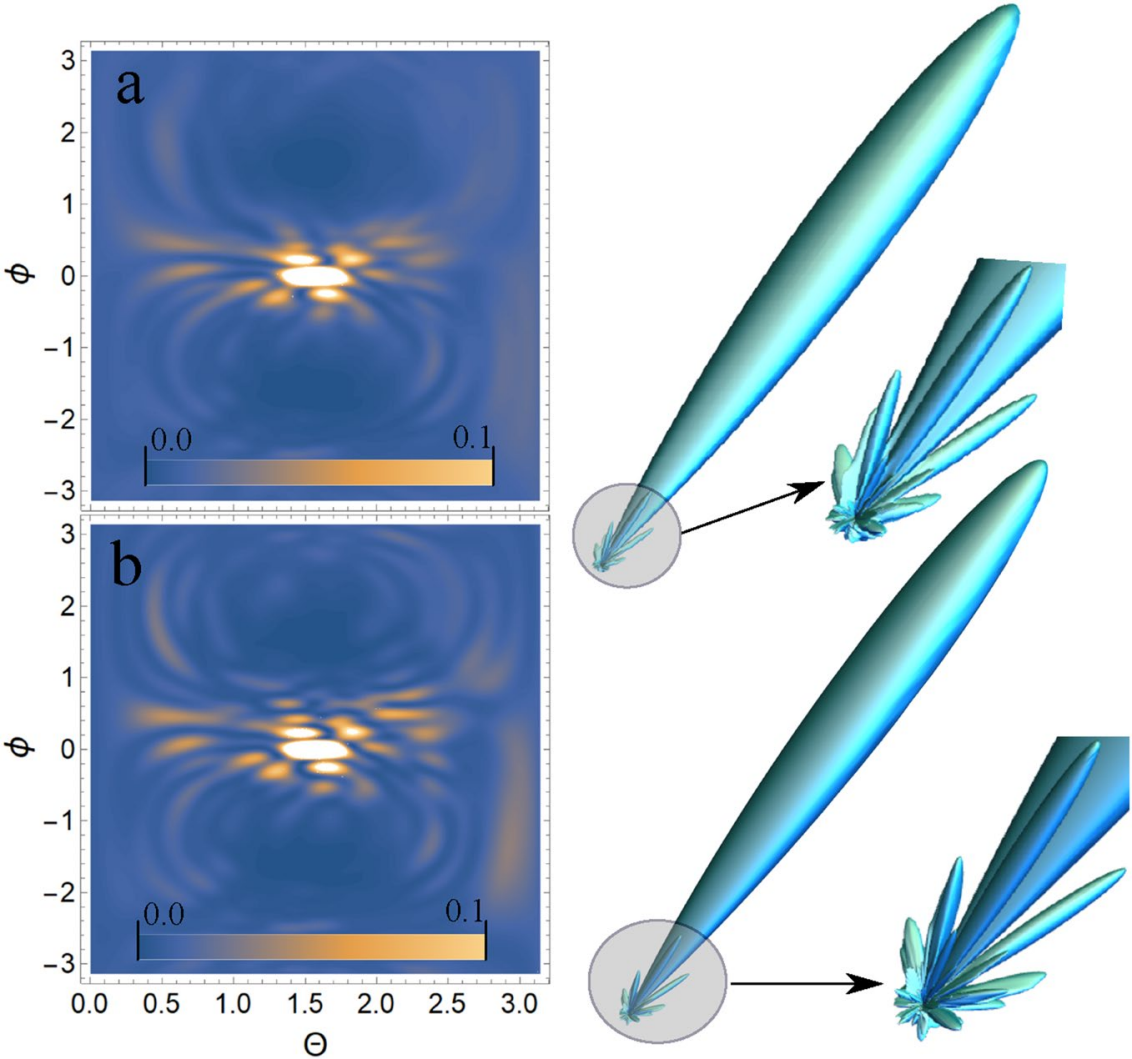
Scattering spectra of USP on guanine are presented. The rest is the same as on Fig. 3.

**Figure 6:**
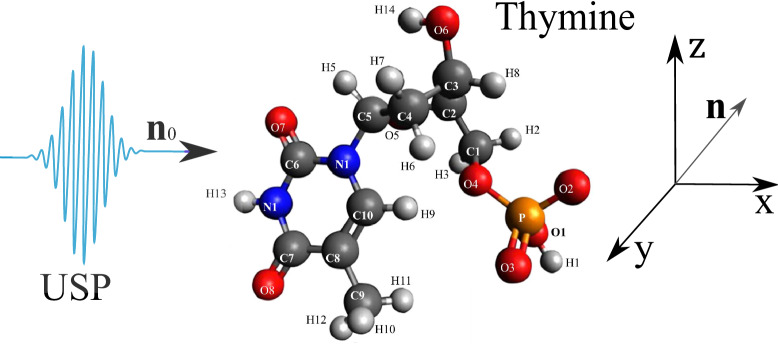
The thymine on which the USP falls is represented, as well as the chosen coordinate system. The calculation was performed in the spatial orientation of the thymine in relation to the USP in the chosen coordinate system shown in this figure.

**Figure 7:**
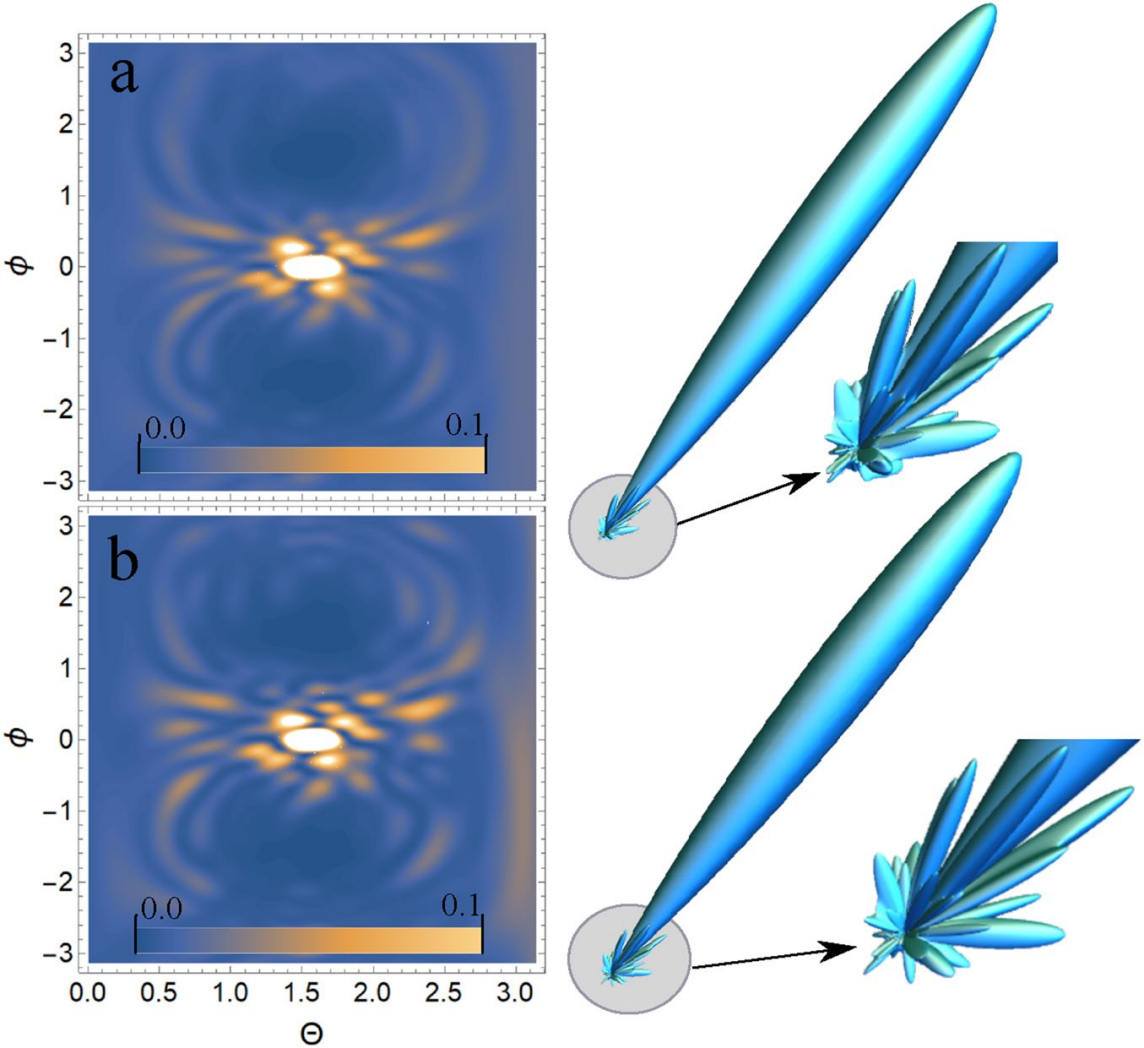
Scattering spectra of USP on thymine are presented. The rest is the same as on Fig. 3.

**Figure 8:**
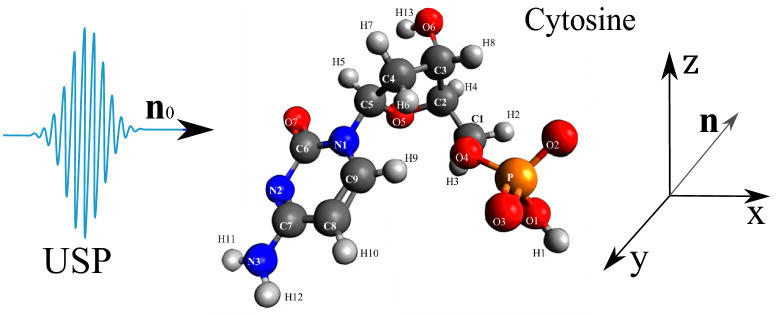
The cytosine on which the USP falls is represented, as well as the chosen coordinate system. The calculation was performed in the spatial orientation of the cytosine in relation to the USP in the chosen coordinate system shown in this figure.

**Figure 9:**
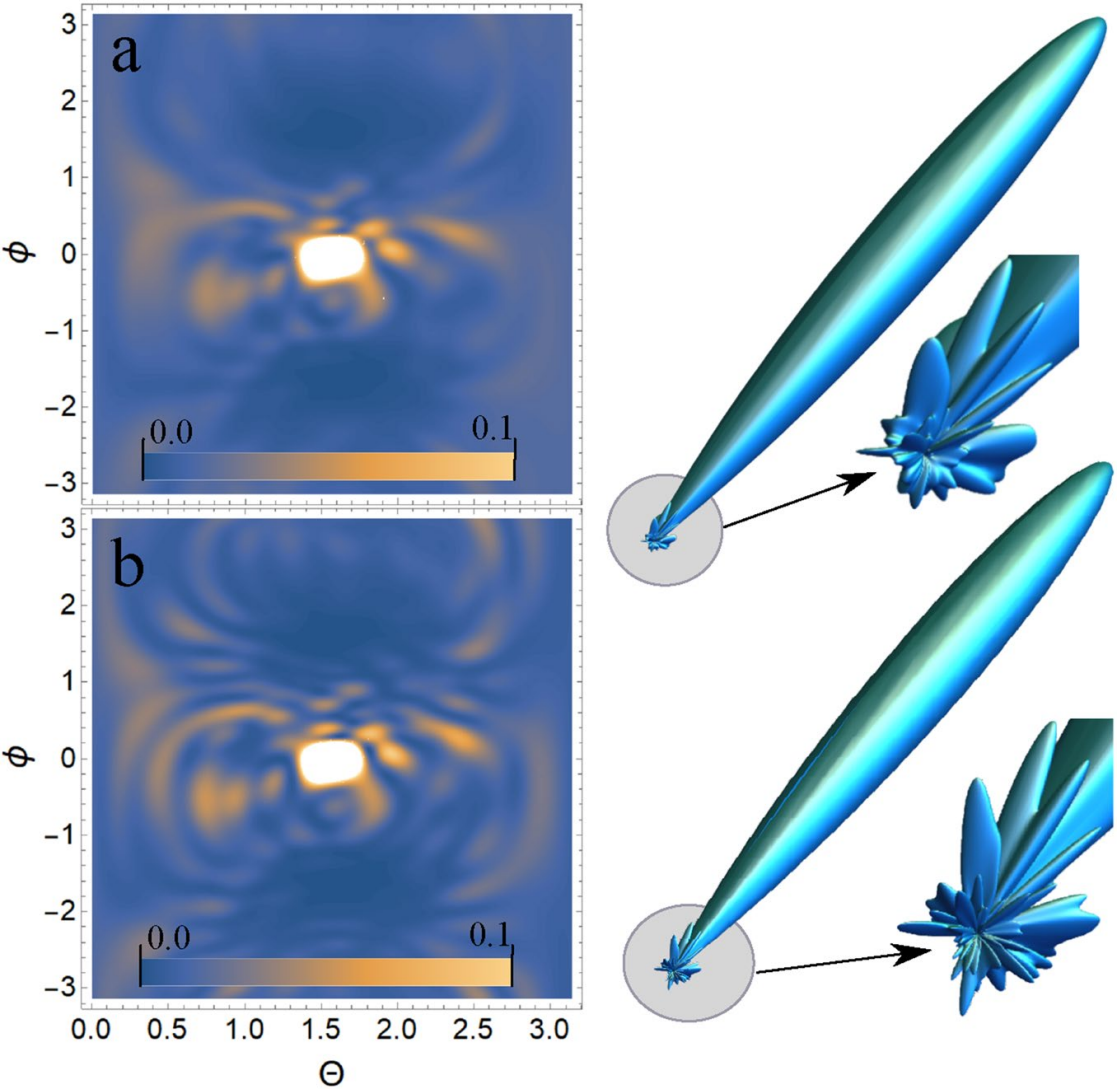
Scattering spectra of USP on cytosine are presented. The rest is the same as on Fig. 3.

Let us show and compare the scattering spectra using the well-known XRD theory (see Eq. (5)) and the theory presented here (see Eq. (4)) at $\tau \omega _0\gg 1$. On Fig. 2, 3, 4, 5, 6, 7, 8 and 9 shows the results of calculations of USP scattering on nucleotides: adenine, see Fig. 2 and for scattering spectra, see Fig. 3; guanine, see Fig. 4 and for scattering spectra, see Fig. 5; thymine, see Fig. 6 and for scattering spectra, see Fig. 7; cytosine, see Fig. 8 and for scattering spectra, see Fig. 9. The calculation results in all figures are normalized to the maximum value of the scattering spectrum. The value is $\omega _0=2c$, and $\alpha =c/4$, which corresponds to the photon energy $\hbar \omega _0=7.46$ *keV*. The choice of intensity USP $I\propto E^2_0$ does not affect the spatial distribution of the scattering intensity, so these parameters can be omitted (taking into account the chosen normalization to the maximum value of the scattering spectrum). All presented figures show the scattering spectra depending on the direction of the scattered USP $\mathbf{n}$. It should be added that scattering spectra are often presented in the literature as functions of the wave vector $\mathbf{p}=\omega _0/c (\mathbf{n}-\mathbf{n}_0)$. We will present the scattering depending on the direction of $\mathbf{n}$, since in our theory calculations there is one more vector $\mathbf{p}_{\tau }=\frac{1}{c\tau } (\mathbf{n }-\mathbf{n}_0)$. It is obvious that only the vector $\mathbf{n}$ is a variable in scattering, all other parameters are specified, so our representation is more convenient. Although it can also be represented in terms of the $\mathbf{p}$ vector, this will complicate the analysis and interpretation of the results obtained. It should be added that there is no point in presenting the results of the calculations in absolute intensity units, because the maximum value of the scattered USP can easily be obtained from Eq. (7) and will be $\left( \frac{d\varepsilon }{d\Omega _\mathbf{k}}\right) _{max}=\frac{\left[ \mathbf{E}_0\mathbf{n}\right] ^2}{2 c^3 \alpha \sqrt{2\pi }} N^2$, where *N* is the number of electrons in the multi-atomic molecule in question. Therefore, it is easier to normalize the calculation results to this maximum value, i.e. the figures will show the results of calculations $\left( \frac{d\varepsilon }{d\Omega _\mathbf{k}}\right) /\left( \frac{d\varepsilon }{d\Omega _\mathbf{k} }\right) _{max}$.

In all presented figures (a) and (b), one can see the same differences in the scattering spectra. First, at sufficiently large scattering angles $\theta$ and $\phi$, one can see significant differences in the spectra in all Fig. (a) and (b). This is due to the fact that large scattering angles $\theta$ and $\phi$ are responsible for scattering (more precisely, for diffraction) not only between closely spaced atoms in a nucleotide, but also distant from each other. When the atoms are far enough apart, the expression $e^{-\frac{1}{2} (\mathbf{p}_{\tau } (\mathbf{R}_{Ai}-\mathbf{R} _ {A^{'}j}))^2}\ll 1$ shows that diffraction does not matter at such interatomic distances. This fact is reflected in figures (a), whose spectra were calculated from Eq. (4). Secondly, at sufficiently small scattering angles $\theta$ and $\phi$ the spectra are close to each other in Fig. (a) and (b). This can be easily explained if we see that at small scattering angles the quantity $\mathbf{p}_{\tau } (\mathbf{R}_{Ai}-\mathbf{R}_{A^{'}j} )$ is small, and hence the results of the calculation by Eqs. (4) and (5) will be close. It should be added that these are the results of calculations at the selected parameters of pulse duration $\tau$ and carrier frequency $\omega _0$. If you choose $\omega _0$ more, the diffraction pattern will be more diverse, because in this case there will be more diffraction maxima. If a smaller value of $\tau$ is chosen, the differences in the calculations of Eqs. (4) and (5) will be more significant, since the parameter $\beta _{i,j}(\mathbf{p}_{\tau })$ becomes more sensitive. The directions of the smaller peaks in all of the figures are complex and are set by the spatial arrangement of the atoms in a given molecule. The arrangement of the small peaks is asymmetric, which is due to the asymmetric arrangement of the atoms in the nucleotides. One can also see that, in general, the direction and size of most of the scattered peaks are located in the direction of the incident pulse.

## Discussion and conclusion

Thus, we obtained a general Eq. (3) for calculations of scattering spectra of USP on complex polyatomic structures. The main value responsible for the spatial arrangement of atoms in the system is determined by the parameter $\delta _{i,j}$, calculated by the Eq. (6). In the case of multi-cycle USP Eq. (3) can be represented as Eq. (4), and in the case of a multicycle Gaussian pulse, one can represent Eq. (7). The obtained expressions have an analytical form, which greatly simplifies the calculations and the interpretation of the results obtained. One important consequence of the theory developed here is that it differs significantly from the well-known XRD theory (see Eq. (5) for attosecond USPs and polyatomic molecules in the case of multi-cycle pulses. It should be added that multi-cycle pulses are currently used for XRD. In other cases, it makes no sense to compare the theory developed here with Eq. (5), since it is obvious that in the case of low-cycle pulses the differences will be large. The scattering, on the nucleotides considered, of the attosecond USP differs from the known XRD theory (see Eq. (5)). This is certainly an important result, since the study of DNA and RNA using modern USP sources (e.g., XFELs) is one of the most promising areas of science. For systems consisting of a small number of atoms, Eq. (3) must be used since the incoherent part in the scattering spectrum makes a significant contribution. This is easily shown using Eq. (7) at $\lambda _0 \ll 1$, then $\left( \frac{d\varepsilon }{d\Omega _\mathbf{k}}\right) _{max} \sim N_e+N ^2_e (\lambda _0/a)^4$, where $a\sim 1$ and $N_e$ is the total number of electrons in multi-atomic structures. For $\lambda _0 \gg 1$ it turns out that $\left( \frac{d\varepsilon }{d\Omega _\mathbf{k}}\right) _{max} \sim N_e+N^2_e$. This is an important refinement because the incoherent part in the scattering spectra of X-ray USPs is usually not taken into account.

The theory developed here is primarily important for XRD using attosecond pulses and polyatomic molecules (various macromolecules, biomolecules, including DNA and RNA, etc.). Because it is in this case that the theory presented here differs from the previously known theory of XRD. In the case of multi-cycle and long duration pulses (many times more attosecond pulses) and polyatomic molecules, the theory presented here coincides with the previously known XRD theory. It should be added that the theory presented here extends to USPs of any duration in the X-ray frequency range, with the exception of much shorter than attosecond ones, i.e. the theory presented here is correct for pulse duration $\tau \gg 1/c^2$ ($\tau \gg 8.1\times 10^{-21} s.$).
